# Spontaneous decompression of sigmoid volvulus

**DOI:** 10.12669/pjms.39.6.8052

**Published:** 2023

**Authors:** Nurhak Aksungur, Rifat Peksoz, Esra Disci, Sabri Selcuk Atamanalp

**Affiliations:** 1 Nurhak Aksungur, MD Assistant Professor, Department of General Surgery, Faculty of Medicine, Ataturk University, Erzurum, Turkey; 2 Rifat Peksoz, MD Assistant Professor, Department of General Surgery, Faculty of Medicine, Ataturk University, Erzurum, Turkey; 3 Esra Disci, MD Associate Professor, Department of General Surgery, Faculty of Medicine, Ataturk University, Erzurum, Turkey; 4 Sabri Selcuk Atamanalp, MD Professor, Department of General Surgery, Faculty of Medicine, Ataturk University, Erzurum, Turkey

**Keywords:** Sigmoid volvulus, Spontaneous, Decompression

## Abstract

**Objectives:**

Spontaneous decompression is an uncommon outcome of sigmoid volvulus (SV). The aim of this study was to evaluate the clinical presentation, diagnosis, treatment, and follow-up of spontaneously decompressed SV.

**Methods:**

We utilized the data of our 1,063 SV patients, the most comprehensive monocenter SV series in the world. To obtain the worldwide data on the spontaneous decompression of SV, we researched the last 56-years’ literature in Web of Science and PubMed databases.

**Results:**

The incidence of the spontaneous decompression was 0.1% (1/1,063) in our SV series, whereas it was 1.5% (8/549) in the worldwide data (Fisher exact test, p = 0.001). By this way, cumulative spontaneous decompression rate was found as 0.6% (9/1,602). In the spontaneously decompressed cases, the main clinical features were abdominal pain/tenderness, distention, and obstipation, which were similar to management-required patients. However, the treatment and follow-up algorithm is still a relatively undefined subject.

**Conclusion:**

Spontaneous decompression of SV is a very rare clinical entity. The clinical presentation and diagnosis of the spontaneously decompressed SV look alike the management-required SV. However, as seen in most management-required patients, SV tends to recur in the spontaneously decompressed cases and a recurrence-reducing procedure is required in selected patients.

## INTRODUCTION

Spontaneous decompression is an unusual outcome of sigmoid volvulus (SV), the rotation of the sigmoid colon around itself causing an obstruction in colonic passage.^1^,^2^ Although SV typically ranks among the literature knowledge related to intestinal system, its spontaneous decompression is a relatively unclarified subject due to its rarity.^3^,^4^ Ataturk University Faculty of Medicine is the biggest regional health organization in Eastern Anatolia, an endemic area for SV. This medical center has 1,063-patient experience with SV over 56.5-year period from June 1966 to January 2023. This is the most comprehensive monocenter SV series over the world.^5^,^6^ For this reason, we wanted to share our opinion and clinical experience on the spontaneous decompression of SV.

## METHODS

In our clinic, total 1,063 SV cases were treated during 56.5-year period from June 1966 to January 2023. Among them, the clinical data including treatment options of 612 patients (58.2%) were investigated retrospectively till June 1986, whereas 439 (41.8%) cases were evaluated prospectively thereafter. Following diagnosis and resuscitation, patients were treated with endoscopic decompression (those without bowel gangrene or peritoneal irritation) or emergency surgery (those with above-mentioned adverse situations or unsuccessful endoscopic decompression), while a case was spontaneously decompressed under medical observation in preoperational period.

To obtain the worldwide data on the spontaneous decompression, an investigation was performed in Web of Science^3^ and PubMed^4^ databases under the head of ‘sigmoid volvulus’ and related results were utilized together with our findings. To compare our results with worldwide data on the spontaneously decompressed SV, a statistical analysis was performed by using SPSS v22.0 system (IBM Corporation, Armonk, New York, United States). Categorical variables were compared by using Chi-Square or Fisher exact tests. Significance level was set up p<0.05.

### Ethical approval

Ethical approval was obtained from Ethical Committee of Ataturk University Faculty of Medicine (No: 88-2022). Written informed consent for scientific research was obtained from all participants.

## RESULTS

In our 1,063-case series, spontaneous decompression was demonstrated in one patient (0.1%). As worldwide data on the spontaneous decompression of SV, among total 1,209 publications, 671 papers with undetailed data were excluded and the remained 538 articles were investigated. However, as demonstrated in Table-I, only four articles involving detailed data were found on this subject.^3^,^4^ Among them, in a relatively large series including 396 patients, Pattanaik^1^ notified 0.5% of spontaneous decompression rate for SV, while this rate was reported as 3.0% (3/99) by Arnold and Nance,^7^ 4.0% (1/25) by String and DeCosse,^8^ and 6.9% (2/29) by Lau et al.^9^ Accordingly, the spontaneous decompression of SV was found to be statistically more common in worldwide data when compared with our results (1.5%, 8/549 vs. 0.1%, 1/1,063, respectively, Fisher exact test, p = 0.001). On the other hand, when our results were evaluated together with worldwide data, the spontaneous decompression rate was demonstrated as 0.6% (9/1,602).

**Table-I:** Spontaneous decompression of sigmoid volvulus in worldwide literature.

Author	Year	Sigmoid volvulus	Spontaneous decompression	%
String and DeCosse^8^	1971	25	1	4.0
Arnold and Nance^7^	1973	99	3	3.0
Lau et al^9^	2006	29	2	6.9
Pattanaik^1^	2018	396	2	0.5
Worldwide data		549	8	1.5
Our series	2023	1,063	1	0.1

Total		1,612	9	0.6

In detailed evaluation, the main clinical features of the spontaneously decompressed cases were not different from that of the management-required patients, which mainly included abdominal pain/tenderness, distention, and obstipation. Similarly, the main diagnostic procedures were plain abdominal X-ray radiograms in addition to computed tomography (CT) in recent years (1/9, 11.1%). Unfortunately, there was no information about add-on-therapy in cases reported in the literature, whereas percutaneous endoscopic colopexy (PEC) was suggested in our case, whose heavy physical status (American Society of Anesthesiologists-ASA score 4) didn’t allow an elective sigmoid colectomy.

## DISCUSSION

In SV, the spontaneous decompression rate may rise up to 44.1% depending on the anamnesis of the patients.^10^ This relatively high rate, which doesn’t reckon with diagnostic accuracy, may be explained by incomplete volvulus attacks arising from partial torsion of the sigmoid colon (≤180^o^) and such movements are accepted as physiological rotations, which generally resolve spontaneously.^11^ However, the actual rate of the spontaneous decompression following clinical diagnosis and under medical observation in a healthcare organization is quite low, which can be expected in about 2% of cases.^10^

Hence, our search of the last 56-years’ literature from 1967 to date in Web of Science^3^ and PubMed^4^ databases demonstrated no more than a handful of relevant articles, in which the spontaneous decompression rate was reported to be between 0.5% and 6.9%.^1^,^7^-^9^ In our series, this rate was 0.1% (1 of 1,063 cases). As a result, when the diagnosis of SV in a health center is considered, total number of the reported patients with spontaneous decompression can be counted on the fingers of two hands.

Most likely due to the rarity of the spontaneous decompression, its pathophysiology is not clearly identified in the literature.^1^,^3^,^4^,^7^-^9^ As known, the most important anatomical prerequisite is dolichosigmoid (an elongated and dilated sigmoid colon with a long mesentery) in the development of both primary and recurrent SV.^12^,^13^ In our opinion, repetitive SV attacks may cause mesenteric fibrosis, which forms a narrow-based mesentery resulting in both easy recurrence and the spontaneous decompression. Although there is no information about the pathophysiology of above-mentioned cases reported in the literature, at least, this theory may apply to our patient.

Although literature findings have no detailed data on the ages and genders of the spontaneously decompressed cases,^1^-^4^,^7^-^9^ all of the reported patients are adults. In our opinion, the presence of a relatively narrow abdominal cavity may make the spontaneous decompression difficult in children. Similarly, due to the similar reason, the spontaneous decompression may be difficult in pregnant women as well as in men, in the last which, a significant high incidence may cause an adverse outcome, as was seen in our case.

**Fig.1:**
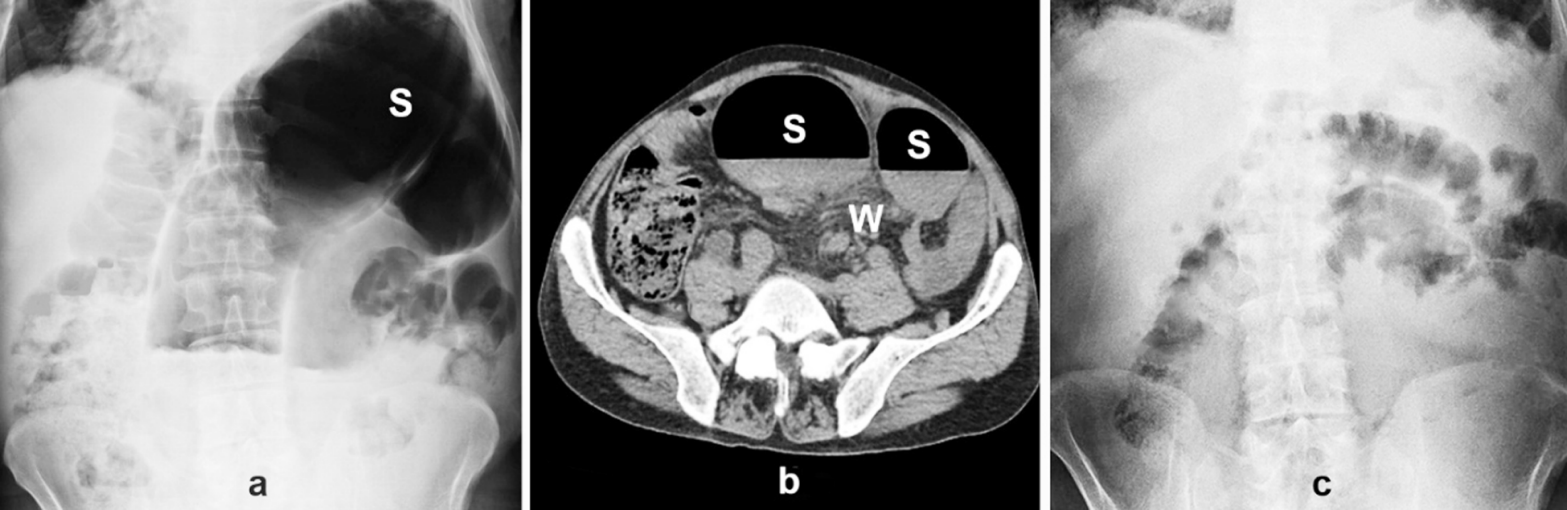
a) Abdominal X-ray radiography image (S: Dilated sigmoid colon) b) Axial abdominal computerized tomography image (S: Dilated sigmoid colon, W: Mesenteric whirl sign) c) Control abdominal X-ray radiography image.

When compared with non-operatively or surgically treated patients, the clinical presentation and diagnosis of SV don’t differ in the spontaneously decompressed cases.^1^,^2^,^7^-^9^ Abdominal pain/tenderness, distention, and obstipation/constipation are the main features.^10^,^14^-^17^ Abdominal x-ray radiography presents a dilated sigmoid colon (Fig.1a), while CT is preferred due to its higher diagnostic value with the same finding in addition to mesenteric whirl sign (Fig. 1b).^10^,^15^,^17^-^19^ In clinical course, defecation including normal-appearing stool, degasification, and abdominal relaxation are the main features of the spontaneous decompression.^2^ Although control CT may demonstrate the disappearance of the previous pathological abdominal findings, control x-ray radiography may also serve the same purpose (Fig.1c).

In the spontaneously decompressed patients, the main treatment rules are also similar to that of the other cases.^1^,^2^,^7^-^9^ Endoscopic decompression is the primary choice in patients without sigmoid gangrene and peritonitis, whereas cases with above-mentioned adverse situations in addition to unsuccessful non-operative treatment requires emergency surgery.^2^,^10^,^15^,^20^-^22^. Regarding the follow-up planning, SV tends to recur in about 25% of the patients and just like the endoscopically decompressed patients, cases with spontaneous decompression are potential candidates for recurrent SV attacks.^2^ For this reason, elective sigmoid colectomy must be suggested in non-elderly and well-conditioned patients, whereas PEC may be an alternative in elderly or bad-conditioned cases.^2^,^10^,^22^-^25^.

### Limitations

Limited number of SV cases with spontaneous decompression in our series and worldwide data in addition to partial retrospective evaluation and long-term study period of our series are probable limitations of this study. However, to obtain sufficient material on SV in a prospective study is quite difficult due to relatively low SV incidence over the world, which obligates the retrospective evaluation of such long-term studies.

## CONCLUSIONS

Spontaneous decompression is a very rare clinical entity, which is seen in about 0.1-6.9% of SV patients. In such cases, the clinical presentation and diagnosis look alike the management-required individuals. However, SV tends to recur in most spontaneously decompressed patients and the most suitable treatment option is to use a recurrence-reducing procedure in selected patients.

### Authors Contribution:

**NA** & **SSA:** Data collection, manuscript writing, revision of the final manuscript.

**RP** & **ED:** Data collection, revision of the final manuscript.

**SSA:** Is responsible for responsible and accountable for the accuracy and integrity of the work.
